# Hepatocyte caveolin-1 modulates metabolic gene profiles and functions in non-alcoholic fatty liver disease

**DOI:** 10.1038/s41419-020-2295-5

**Published:** 2020-02-06

**Authors:** Mei Han, Weronika Piorońska, Sai Wang, Zeribe Chike Nwosu, Carsten Sticht, Shanshan Wang, Yan Gao, Matthias Philip Ebert, Steven Dooley, Christoph Meyer

**Affiliations:** 1 Department of Medicine II, Section Molecular Hepatology, Medical Faculty Mannheim, Heidelberg University, Theodor-Kutzer-Ufer 1–3, 68167 Mannheim, Germany; 2 Department of Internal Medicine, the Second Hospital of Dalian Medical University, Shahekou District, Dalian City, Liaoning Province China; 3 Medical Research Center, Medical Faculty Mannheim, Heidelberg University, Theodor-Kutzer-Ufer 1–3, 68167 Mannheim, Germany; 4 Beijing Institute of Hepatology, Beijing You’ An Hospital Affiliated to Capital Medical University, 100069 Beijing, China

**Keywords:** Mechanisms of disease, Mechanisms of disease, Mechanisms of disease, Transcriptomics, Transcriptomics

## Abstract

Caveolin-1 (CAV1) is a crucial regulator of lipid accumulation and metabolism. Previous studies have shown that global *Cav1* deficiency affects lipid metabolism and hepatic steatosis. We aimed to analyze the consequences of hepatocyte-specific *Cav1* knockout under healthy conditions and upon non-alcoholic fatty liver disease (NAFLD) development. Male and female hepatocyte-specific *Cav1* knockout (HepCAV1ko) mice were fed a methionine/choline (MCD) deficient diet for 4 weeks. MCD feeding caused severe hepatic steatosis and slight fibrosis. In addition, liver function parameters, i.e., ALT, AST, and GLDH, were elevated, while cholesterol and glucose level were reduced upon MCD feeding. These differences were not affected by hepatocyte-specific *Cav1* knockout. Microarray analysis showed strong differences in gene expression profiles of livers from HepCAV1ko mice compared those of global Cav1 knockout animals. Pathway enrichment analysis identified that metabolic alterations were sex-dimorphically regulated by hepatocyte-specific CAV1. In male HepCAV1ko mice, metabolic pathways were suppressed in NAFLD, whereas in female knockout mice induced. Moreover, gender-specific transcription profiles were modulated in healthy animals. In conclusion, our results demonstrate that hepatocyte-specific *Cav1* knockout significantly altered gene profiles, did not affect liver steatosis and fibrosis in NAFLD and that gender had severe impact on gene expression patterns in healthy and diseased hepatocyte-specific *Cav1* knockout mice.

## Introduction

Non-alcoholic fatty liver disease (NAFLD) is a common metabolic liver disorder, characterized by excessive deposition of intrahepatic fat^1^. The incidence of NAFLD has increased in recent years and thus, NAFLD is considered a new societal challenge, requiring novel strategies for disease prevention and treatment^2^. NAFLD encompasses two histological subtypes: non-alcoholic fatty liver characterized by simple liver steatosis and non-alcoholic steatohepatitis (NASH), which describes hepatic steatosis and inflammation with fibrosis. NASH patients have a high risk of developing cirrhosis^3,4^. For a deeper understanding of the disease and for developing therapy, sophisticated NAFLD animal models have been developed. The commonly used models are diverse high caloric diets (e.g., Western diet) and the methionine/choline deficient (MCD) diet. The MCD diet leads to impairment of the secretion of hepatic very low density lipoproteins (VLDL), due to the absence of phosphatidylcholine precursors^5^.

Caveolin-1 (CAV1) is the main structural protein of caveolae—omega-shaped invaginations of plasma membrane defining a specific endocytic route. CAV1 regulates cholesterol transport and membrane distribution, as well as diverse signaling pathways^6^. Moreover, CAV1 is a regulator of metabolism, effecting lipid and glucose metabolism, insulin resistance-, and diet-induced obesity^7–10^. These phenomena are supported by findings in global *Cav1* null mice, that present with strongly elevated triglyceride and free fatty acid levels, and which are resistant to high fat diet-induced obesity^11^. It can also be inferred that CAV1 is an essential factor in lipid regulation during liver regeneration after partial hepatectomy, given that lipid droplet accumulation was reduced and cell cycle was impaired in hepatocytes of global *Cav1* null mice^12^. In another study, *Cav1* null mice displayed decreased adiponectin abundance and reduced metabolic flexibility under fasting conditions. This influenced hepatic steatosis, thus arguing for a non-hepatic CAV1 control of liver metabolic alterations^13^. This prompted us to perform an in-depth study to clarify hepatocyte-specific functions in healthy liver and NAFLD and focused on whether hepatocyte-specific CAV1 deficiency alters metabolic processes in healthy and diseased livers. We additionally aimed to determine gender influence on CAV1 functions, given that males have a higher susceptibility for developing NAFLD in mice and human^14,15^. Interestingly, CAV1 and sex hormones are interacting to interfere with metabolic processes by regulating hormone signaling and altering distinct hormones or hormone receptors^16^.

We report that hepatocyte-specific CAV1 does not affect liver steatosis and fibrosis in the MCD induced NAFLD model, but impacts severely on gene expression profiles, especially in diseased livers of males and females.

## Results

### Hepatocyte-specific *Cav1* knockout in mice

**Fig. 1:**
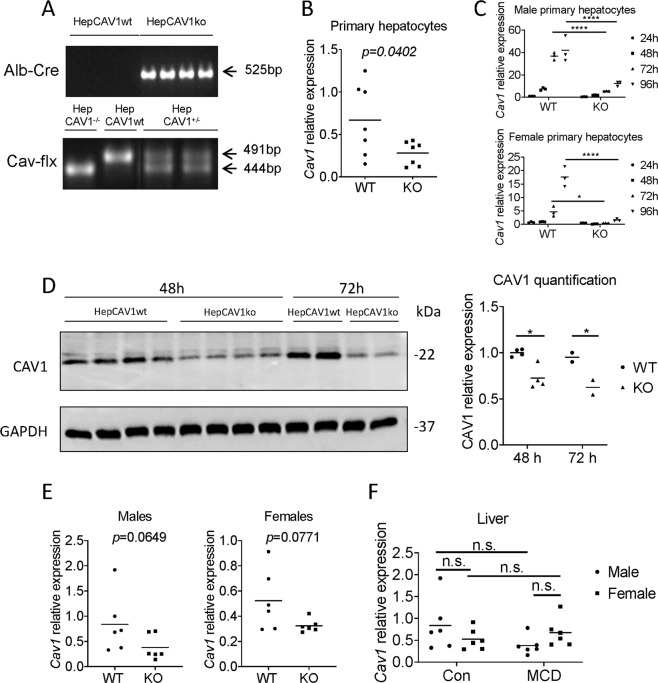
Knockout of hepatocyte-specific *Cav1* in mice. **a** PCR based genotyping of mice including Cre-recombinase test and Cav-flx test. HepCAV1ko mice showed one Alb-cre positive band. Wild-type mice showed one band at 491 bp, and HepCAV1^+/−^ mice showed two bands of sizes at 444 bp and 491 bp, and HepCAV1^−/−^ showed one band at 444 bp. **b** mRNA expression level of *Cav1* in isolated primary hepatocytes from males showed a significant difference between HepCAV1ko and HepCAV1wt (*N* = 7), *p* = 0.0402 using *t*-test. **c** Time course of mRNA expression of *Cav1* in primary hepatocytes isolated from male and female wild-type and HepCAV1^−/−^ mice. In the wild-type mice, *Cav1* is strongly induced over time. **d** Protein expression of CAV1 in isolated primary hepatocytes (males) after 48 h and 72 h cell culture and densitometric analysis of expression intensity. **e** mRNA levels of *Cav1* in liver tissue of males (*N* = 6) and females (*N* = 6) showed decreased expression in HepCAV1ko mice, with *p* values almost reaching significance (data of males were analyzed by Mann-Whitney, and data of females by *t*-test). **f** *Cav1* expression alteration upon MCD diet (4 weeks; *N* = 6) in male and female mice. Total liver *Cav1* expression of male was higher than female, while no significant change was detected. *Cav1* expression showed a reduction tendency in male wild-type mice, while an increasing tendency was found in females upon MCD diet. Data were analyzed by Two-way ANOVA. WT: HepCAV1wt mice; KO: HepCAV1ko mice; Con: control diet; MCD: MCD diet.

We first confirmed hepatocyte-specific CAV1 deletion by genotyping for *Cre* recombinase and *Cav*1 loxp sites (Fig. 1a). In our studies, homozygous HepCAV1^−/−^ mice, which were positive for Cre-recombinase (heterozygous), were considered as hepatocyte-specific CAV1 knockouts (HepCAV1ko), whereas those with HepCAV1^−/−^ and negative Cre-recombinase were used as hepatocyte-specific CAV1 wild-type (HepCAV1wt, i.e., as reference control group).

To test CAV1 expression in hepatocytes, mRNA level of *Cav1* in freshly isolated hepatocytes (*N* = 7) was measured. As expected, *Cav1* was significantly reduced (*p* = 0.0402) in HepCAV1ko compared to HepCAV1wt mice (Fig. 1b). However, due to the low basal expression of CAV1 in primary hepatocytes, CAV1 was not detected by Western blot. Hence, an alternative system to confirm knockout efficiency at protein level was used. Specifically, CAV1 expression of primary hepatocytes is induced over time when cultured in vitro^17^. Hence, we cultured primary cells up to 4 days and harvested RNA and protein lysates at 48 h (*N* = 4) and 72 h (*N* = 2). *Cav1* increased over time in wild-type males and females (Fig. 1c), but only very weak in HepCAV1ko hepatocytes. CAV1 protein expression was significantly higher in HepCAV1wt at both time points, as assessed upon quantification (Fig. 1d). In HepCAV1wt mice, CAV1 abundance increased over time, as expected^17^, whereas no change in HepCAV1ko mice was observed.

With respect to gender, mRNA expression of *Cav1* was obviously reduced in males and females, although *p* values did not reach significance (males: *p* = 0.0649, *N* = 6; females: *p* = 0.0771, *N* = 6, Fig. 1e). It has been reported that high fat diet reduces *Cav1* expression in murine livers^10^. Thus, we checked whether we could recapitulate this finding. The MCD diet led to a reduction of *Cav1* expression in male wild-type mice by tendency, similar as for high fat diet, while an increasing trend was found in females (Fig. 1f). Therefore, a gender-specific regulation seems to occur. Underlining this fact, total liver *Cav1* expression in males was higher than in females, while not being significant (*p* = 0.45, Fig. 1f).

### Lack of hepatocyte-specific CAV1 did not alter body weight and liver/body weight ratio in healthy and NAFLD mice

**Fig. 2:**
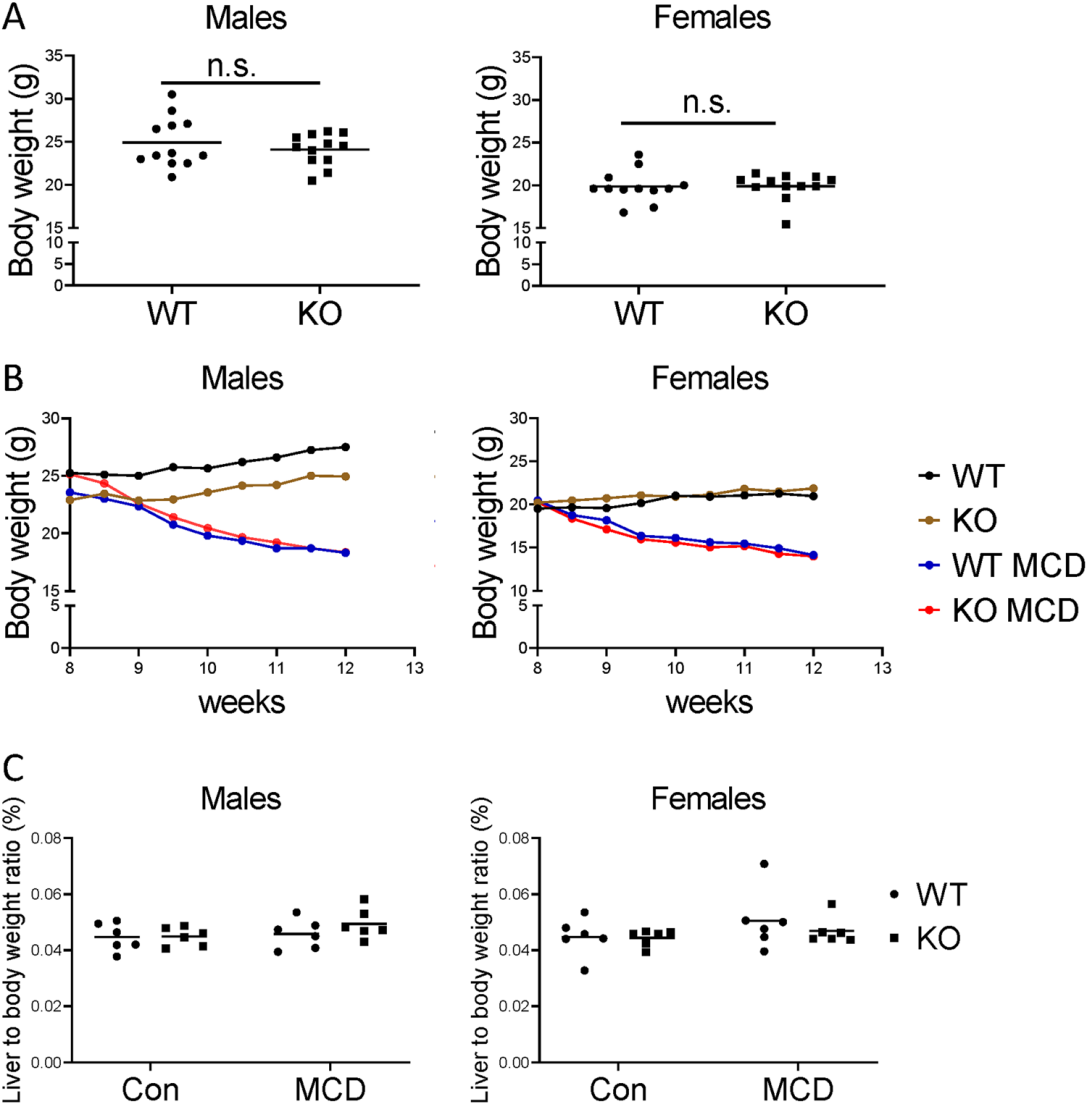
Alteration of body weight and liver/body weight ratio. **a** Body weight comparison between HepCAV1wt and HepCAV1ko mice (*N* = 12). Data of both males and females were analyzed by Mann-Whitney test. **b** Body weight change of males and females during 8th–12th week of experiment. Body weight was significantly reduced upon MCD diet compared with control diet over time, regardless of genotype. **c** Liver/body weight ratio of mice in both genders. No significant differences were determined after MCD feeding by *t*-test. WT: HepCAV1wt mice with control diet; KO: HepCAV1ko mice with control diet; WT MCD: HepCAV1wt mice fed MCD diet; KO MCD: HepCAV1ko mice fed MCD diet.

Prior start of experiments, at the age of 8 weeks, no significant weight difference was observed between HepCAV1wt and HepCAV1ko mice in both sexes (males: HepCAV1wt: 24.92 g ± 2.92 vs HepCAV1ko: 24.09 g ± 1.85; females: HepCAV1wt: 19.88 g ± 1.86 vs HepCAV1ko: 19.88 g ± 1.61; *N* = 12 for each sex, Fig. 2a). Multiple regression analysis showed that gender and diet were significant in body weight change, whereas the genotype and its interactions between gender and diet were not relevant (Fig. 2b). The liver/body weight ratio did not significantly change upon MCD diet in any genotype or any sex (Fig. 2c).

### Hepatocyte-specific *Cav1* knockout does not affect serum parameters

**Fig. 3:**
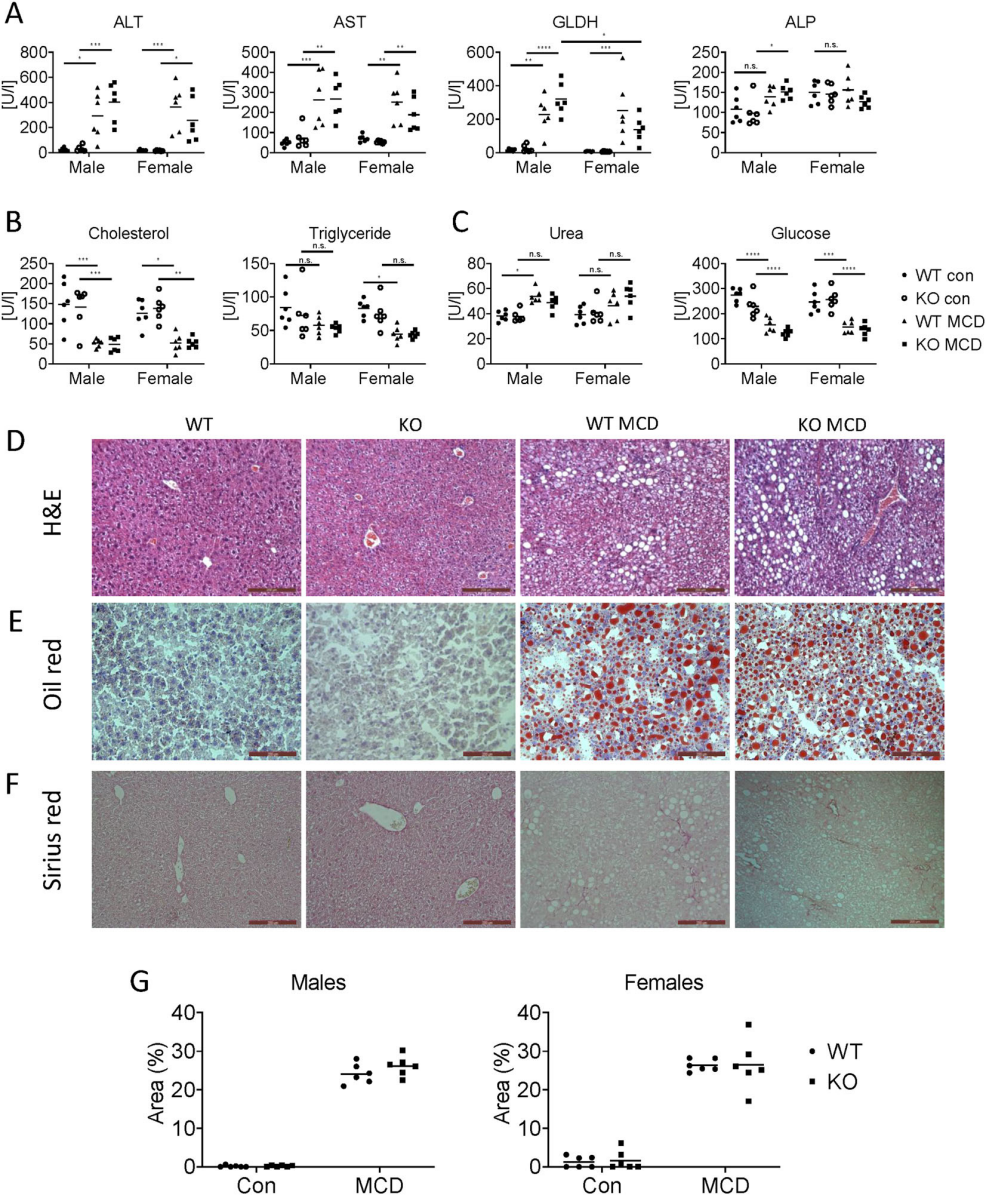
Plasma indices and pathological changes of HepCAV1ko mice upon feeding MCD diet for 4 weeks. **a** Plasma parameters indicating liver injury, i.e., AST, ALT, GLDH, ALP. **b** Lipid metabolism-related parameters, including cholesterol and triglycerides in serum. **c** Carbohydrate metabolism-related parameters, including glucose and urea in serum. **d** H&E staining. **e** Oil Red O staining for testing liver steatosis. **f** Sirius Red staining for detecting fibrosis status. **g** Quantification of Oil Red O staining. *N* = 6 for each group and data was analyzed by ANOVA with multiple comparisons and Tukey’s posthoc test.

To evaluate liver function and integrity, liver injury parameters, such as ALT, AST, GLDH and ALP, were measured (Fig. 3a, see Table 1). Plasma ALT and AST were elevated by MCD diet, but gender and genotype did not show any influence. Alkaline phosphatase did not change significantly in HepCAV1wt mice, while it showed significant difference in HepCAV1ko male mice upon MCD diet. GLDH increased significantly after MCD diet in HepCAV1wt males and females, while considering the knockouts, only in males. Significant differences were additionally detected between HepCAV1ko male and female mice fed MCD diet, i.e., reduced GLDH release in females, which indicated a gender difference in connection with hepatocyte CAV1.

**Table 1:** Plasma parameters in mice. Data was shown as mean ± SD.

	Males				Females			
	WT con	KO con	WT MCD	KO MCD	WT con	KO con	WT MCD	KO MCD
ALT	23 ± 13.78	30 ± 24.07	293.8 ± 186.4	401.5 ± 156.3	18.17 ± 5.46	14.67 ± 3.93	364.5 ± 180.5	257.8 ± 177.4
AST	48.9 ± 15.47	73.17 ± 50.49	262.8 ± 136.8	266.3 ± 98.35	70.83 ± 17.63	53 ± 5.76	251.7 ± 102.7	188.3 ± 84.53
ALP	107.8 ± 33.43	98.17 ± 35.44	139.2 ± 26.45	151.2 ± 18.19	150.3 ± 28	146 ± 24.13	156.2 ± 38.82	126.7 ± 15.82
GLDH	15.83 ± 6.97	23.75 ± 21.68	228.2 ± 105.8	320.3 ± 96.61	7.552 ± 2.05	6.29 ± 2.46	251.2 ± 182	138.4 ± 77.43
Cholesterol	148 ± 57.78	141.2 ± 52.49	51.67 ± 10.07	48.17 ± 17.96	126.2 ± 34.39	138.2 ± 31.68	51.67 ± 22.6	52.67 ± 12.16
Triglycerides	84.33 ± 28.32	72.17 ± 36.14	57.67 ± 14.22	53.17 ± 6.31	82.83 ± 12.91	74.33 ± 22.39	44.17 ± 11.62	43.67 ± 5.05
Urea	38.12 ± 4.054	37.8 ± 4.56	53.78 ± 5.44	48.77 ± 5.94	39.13 ± 6.99	40.95 ± 8.75	46.32 ± 11.03	53.92 ± 9.96
Glucose	273.8 ± 27.01	228.7 ± 44.8	155 ± 29.26	123 ± 16.69	246.3 ± 44.2	256.3 ± 41.23	146.2 ± 24.85	135.5 ± 24.44

Regarding metabolic activity, serum cholesterol, and triglyceride levels were measured (Fig. 3b). Serum cholesterol showed a significant reduction in both genders and genotypes upon MCD feeding. Serum triglyceride level only significantly reduced in HepCAV1wt female mice fed MCD compared to controls, but not observed in the other groups (HepCAV1wt male, HepCAV1ko female and HepCAV1ko male mice). Urea and glucose, important indicators of detoxification and glucose metabolism, respectively, were also tested (Fig. 3c). Urea increased significantly in HepCAV1wt male mice after feeding MCD diet, while in all other groups upon MCD feeding, urea was only slightly increased only. Next, glucose itself was significantly reduced upon MCD diet in both genders and genotypes. However, these differences were independent of CAV1 abundance.

### Histology of HepCAV1wt and HepCAV1ko mice fed MCD diet

Complementing findings on CAV1 functions on serum parameters and liver/body weight, architectural changes of NAFLD mouse livers were to be analyzed. Thus, HepCAV1wt and HepCAV1ko mice were initially tested for hepatic steatosis. Notably, H&E staining and Oil Red O staining revealed severe steatosis with various sizes of lipid deposition in the livers of MCD diet mice (Fig. 3d, e). To assess fibrotic remodelling after NAFLD development, Sirius Red O staining was performed. Four weeks MCD feeding yielded only slight fibrosis (Fig. 3f). Quantification of Oil Red O staining positive area showed no significant difference in HepCAV1ko mice compared to control livers of males and females (Fig. 3g). However, all these alterations were independent of an hepatocyte-specific *Cav1* knockout. Ki67 staining was performed to determine the degree of cell proliferation. No significant differences in Ki67 staining were identified between HepCAV1ko and HepCAV1wt with control or MCD diet (Fig. S1). Hence, the lack of hepatocyte CAV1 is not a critical factor for the development of liver steatosis and fibrosis in the MCD model.

### Minimal overlap between HepCAV1ko and global *Cav1* knockout datasets

**Fig. 4:**
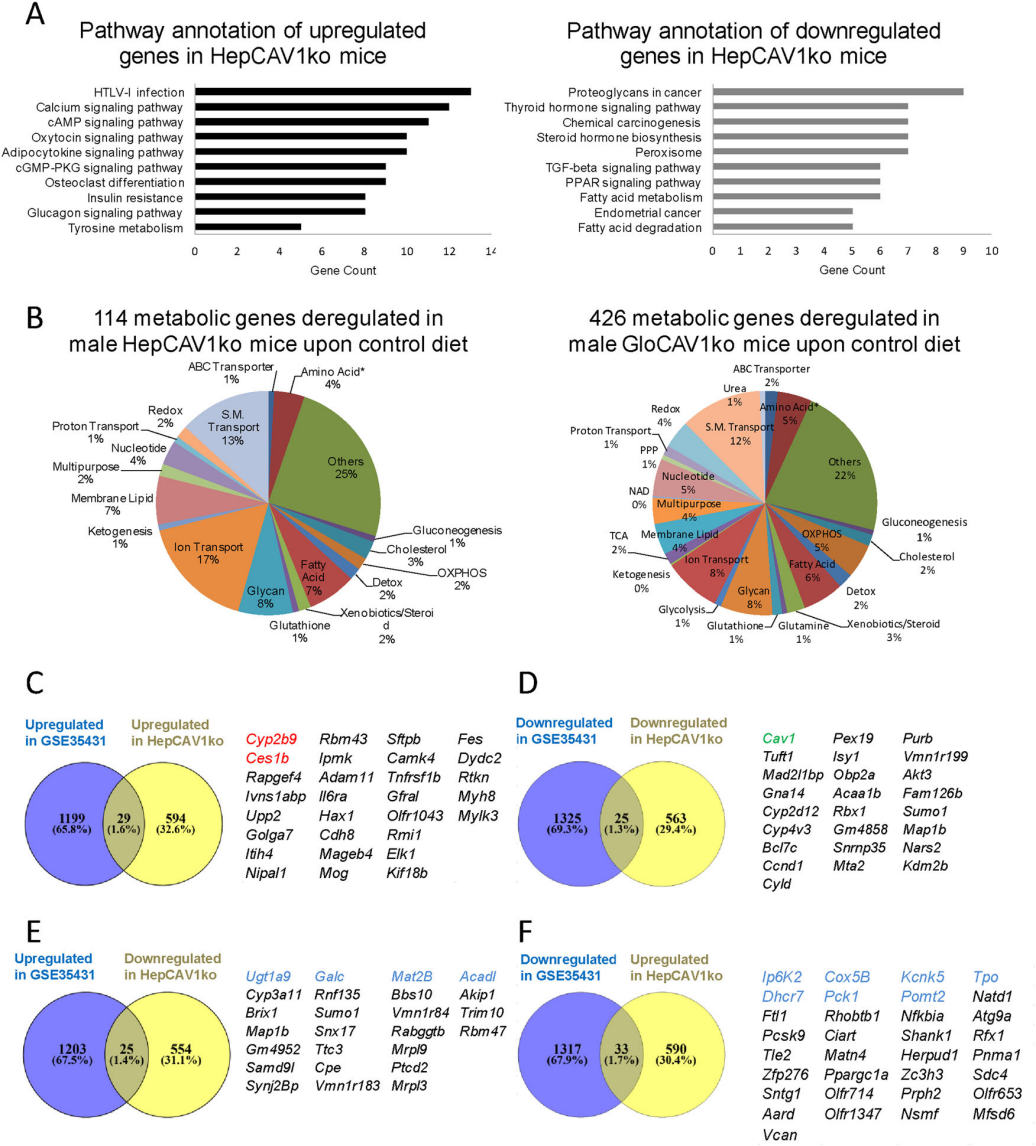
Gene expression profiling of HepCAV1ko and overlap with GloCAV1ko expression data. **a** KEGG pathway annotation of up- or downregulated genes from male HepCAV1ko vs HepCAV1wt mice. **b** Metabolic gene numbers and classification in livers of GloCAV1ko (426 genes) and HepCAV1ko (114 genes) mice. **c** Overlap of upregulated genes in GSE35431 and HepCAV1ko datasets. **d** Distribution of downregulated genes in GSE35431 and HepCAV1ko datasets. **e** Genes upregulated in GSE35431, while inversely expressed in HepCAV1ko dataset. **f** Genes downregulated in GSE35431, while upregulated in HepCAV1ko dataset.

To assess the impact of hepatocyte-specific *Cav1* knockout on liver gene expression profiles, we analyzed microarray data of male HepCAV1ko and HepCAV1wt mice liver. We found 1211 genes regulated (*p* < 0.05), among them 623 upregulated and 588 downregulated genes (Fig. S2A, B, Table S1). KEGG pathway analysis showed that several signaling pathways were deregulated (upregulated genes, connected to e.g., calcium-, adipocytokine-, glucagon-, and oxytocin signaling pathways). Among the downregulated genes, we identified cancer-related signaling pathways, the peroxisome proliferator-activated receptor (PPAR) signaling pathway, the TGF-β signaling pathway and steroid hormone biosynthesis (Fig. 4a, Table S2). Genes in fatty acid β-oxidation and peroxisomal function were also suppressed in gene ontology analysis (Fig. S2C). To compare differences between HepCAV1ko and global *Cav1* knockout (GloCAV1ko), we accessed a published dataset from GloCAV1ko livers (GSE35431, NCBI). Here, we initially analyzed the KEGG pathways and found that both, up- and downregulated genes in GSE35431 were mainly linked with metabolic processes and pathways. Downregulated genes were also related to nervous system diseases, such as Parkinson’s, Huntington’s, and Alzheimer’s disease. Relevant genes are also listed in Table S3. For further comparison of HepCAV1ko and GloCAV1ko datasets in context of metabolic processes, we screened for metabolic genes in each dataset. One hundred and fourteen metabolic genes were deregulated in HepCAV1ko compared to HepCAV1wt mice, while 426 metabolic genes were deregulated in GloCAV1ko compared to GloCAV1wt mice, demonstrating that the global knockout has tremendous more impact on metabolic processes, outperforming HepCAV1ko mice almost 4-fold (Fig. 4b). To summarize, the global absence of CAV1 has stronger impact on metabolic processes than a CAV1 knockout specifically in hepatocytes, indicating that although hepatocytes are the main metabolic cells in the liver, the environment owns potent influence which could, to a significant degree, be CAV1 dependent.

Next, we compared the GloCAV1ko with the HepCAV1ko to assess the degree of overlap. Indeed, only 29 upregulated and 25 downregulated genes overlapped in the two datasets (Fig. 4c, d). Interestingly, there were also some genes showing opposite regulation, as 25 genes were upregulated in GloCAV1ko dataset while downregulated in the HepCAV1ko dataset, and 33 genes were downregulated in GloCAV1ko and upregulated in the HepCAV1ko livers (Fig. 4e, f). Among the overlapping genes, only 13 genes were connected to metabolism (*Cyp2b9, Ces1b, Ugt1a9, Galc, Mat2b, Acadl, Ip6k2, Cox5b, Kcnk5, Tpo, Pomt2, Dhcr7, Pck1*). In summary, there were less than 2% of total genes overlapping and contained only a limited number of metabolic genes. This indicated that the type (hepatocyte-specific vs global) of *Cav1* knockout has distinct consequences on gene expression patterns in mouse livers.

### Gene expression changes upon MCD diet

**Fig. 5:**
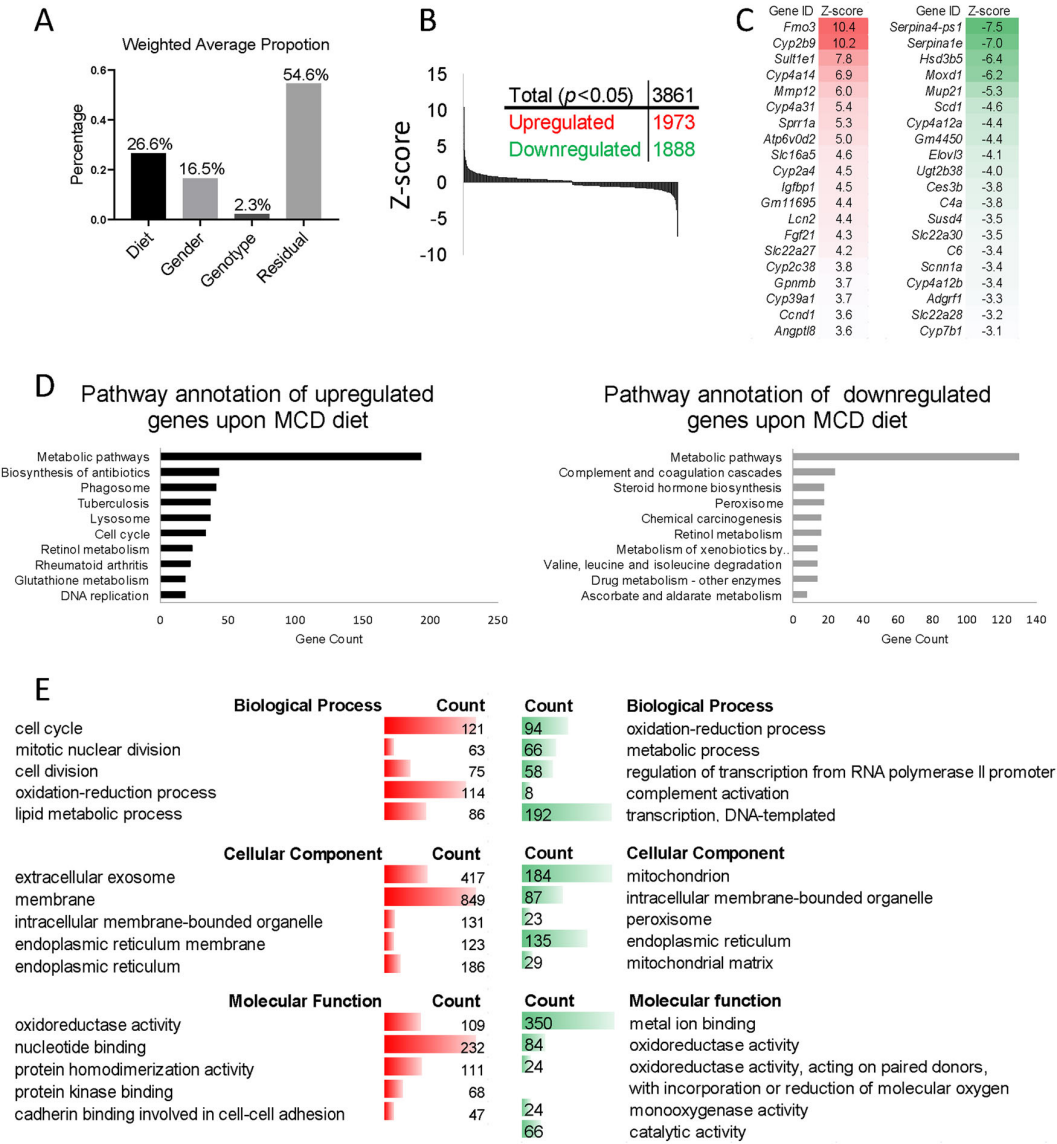
Microarray analysis for MCD diet-induced changes in male HepCAV1wt mice. **a** Principal component analysis revealed the proportion of factors including diet, gender, genotype and residual. **b** Number of differentially regulated genes (*N* = 3861). **c** List of top and bottom 20 significantly regulated genes. **d** KEGG pathway annotation of up- or downregulated genes proves that MCD diet has most impact on metabolic pathways. **e** Molecular functions, biological processes and cellular components analyses of up- or downregulated genes confirmed the results from KEGG pathway annotation. red: upregulated, green: downregulated.

We considered three main features as independent variables in this study, namely: genotype (HepCAV1ko vs HepCAV1wt), diet (MCD vs control diet) and gender (males vs females). Principal component analysis showed that the diet factor accounted for 26.6% of effects, gender for 16.5%, and the genotype solely for 2.3%, whereas residual effects were also strong (54.6%, Fig. 5a). Thus, the impact of the hepatocyte-specific *Cav1* knockout on disease outcome was indeed minor. In contrast, MCD diet, which is a well described NAFLD model strongly altered the gene expression profiles. Initially, expression data from HepCAV1wt male mice that were fed the MCD diet for 4 weeks was analyzed and compared with data from HepCAV1wt male mice fed control diet. 3861 genes were regulated (*p* < 0.05, 1973 upregulated and 1888 downregulated, Fig. 5b, c, Table S4). KEGG pathway annotation indicated that the MCD diet caused overall upregulation of genes related to metabolic pathways and several metabolic processes, including retinol metabolism (e.g., *Cyp2c55, Cyp2b9, Cyp3a11*) and glutathione metabolism (e.g., *Gsta4, Gclc, Ggct*), but also genes linked to DNA replication (e.g., *Lig1, Pole, Pola1*) and cell cycle (e.g., *E2f1, Dbf4, Tgfb3*, Fig. 5d, Table S5). These expression changes were confirmed using gene ontology analysis, which identified several metabolism-related alterations, including oxidation-reduction processes and lipid metabolism and cell cycle (Fig. 5e). However, downregulated genes were also mainly classified to metabolic pathways as analyzed by KEGG, e.g., drug metabolism (e.g., *Upb1, Nat1, Ugt2b1*) and amino acid degradation (e.g., valine, leucine and isoleucine; *Aldh6a1, Acadsb, Acsf3*, Fig. 5d, Table S5). Similar changes were found in gene ontology analysis with respect to metabolic processes, mitochondrial, complement activation and endoplasmic reticulum (Fig. 5e).

### Gender differences in gene expression profiles

**Fig. 6:**
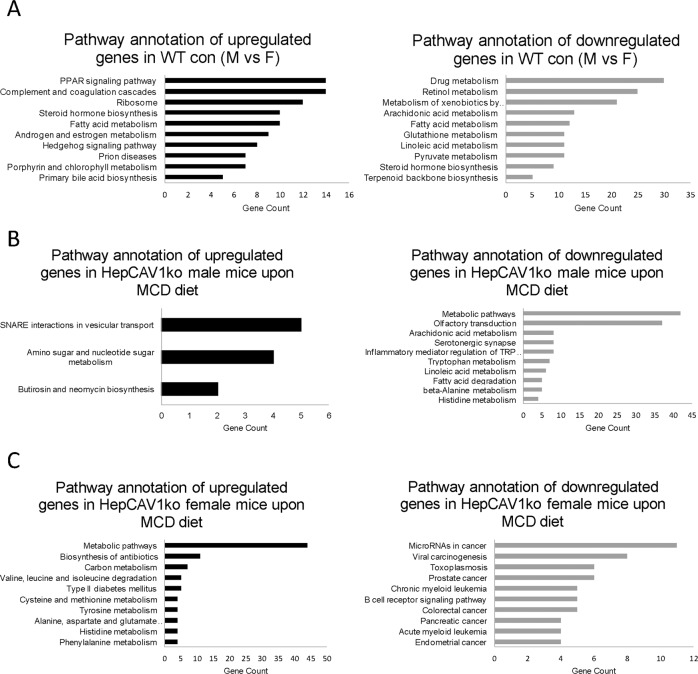
Gender differences of deregulated genes in males compared to females. **a** KEGG pathway annotation of highly or lowly regulated genes comparing males to females in HepCAV1wt mice with control diet. **b** KEGG pathway annotation of up- or downregulated revealed that downregulated genes were related to lipid metabolism after MCD diet in HepCAV1ko compared to HepCAV1wt male mice. **c** KEGG pathway annotation of deregulated genes showed that HepCAV1ko in female induced metabolic processes when compared to HepCAV1wt female mice upon MCD diet. M males, F females.

From phenotypic and microarray principal component analyses, gender was determined as another crucial factor, that exerted strong effects on gene expression changes. Hence, gender differences in HepCAV1wt and HepCAV1ko mice under healthy conditions and NAFLD were investigated. First, we compared gene patterns of HepCAV1wt in healthy males and females to evaluate basal gender differences. Surprisingly, 2618 genes were significantly deregulated between males and females (*p* < 0.05, 1311 higher in males and 1307 higher in females, Tables 2, S6). KEGG pathway analysis showed that genes significantly higher expressed in male mice were related to PPAR signaling, ribosome and various metabolic processes including fatty acid metabolism and steroid hormone biosynthesis. Genes which were significantly more expressed in female mice were also closely related with metabolic processes, such as drug-, retinol-, and glutathione metabolism (Fig. 6a, Table S7). Interestingly, fatty acid metabolism was detected in both upregulated and downregulated gene sets, hence, a reliable conclusion in which direction gender effects are to be interpreted is impossible.

**Table 2:** Number of total regulated genes and metabolic genes in comparison of males and females.

	WT con (M vs F)	KO con (M vs F)	WT MCD (M vs F)	KO MCD (M vs F)
Total deregulated genes	2618	2667	1777	1402
Upregulated genes	1311	1107	951	756
Downregulated genes	1307	1560	826	646
Total deregulated metabolic genes	382	433	204	239
Upregulated metabolic genes	146	136	117	98
Downregulated metabolic genes	236	297	87	141
Metabolic /total deregulated genes (%)	14.59%	16.24%	11.48%	17.05%

Furthermore, gender analyses of healthy and NAFLD HepCAV1ko mice showed that the number of deregulated gene was less upon MCD diet compared to control diet groups of both genotypes (wild-type: 2618 genes vs 1777 genes upon MCD diet, knockout: 2667 genes vs 1402 genes upon MCD diet), which indicated that the diet dominated gender effects (Table 2). From the KEGG pathway analysis for up- or downregulated genes in each group, metabolic reprogramming was shown as a common phenomenon (Fig. S3).

Ratios of metabolic/total deregulated genes of male versus female mice in each group (WT control diet—14.59%; KO control diet—16.24%; WT MCD diet—11.48%; KO MCD diet—17.05%) were between 11–18%. To further evaluate the gender difference caused by *Cav1* knockout mice between wild-type and knockouts, we compared overlapping genes after normal diet. Here, 126 genes were downregulated (20.3%) and 60 upregulated (9.7%) in both dataset (Fig. S4A, Table S8). After MCD diet, only 16 genes showed a downregulation (4%) and 20 were upregulated (5%, Fig. S4B, Table S8). Thus, the gender-dependent differences caused by *Cav1* knockout in metabolic gene expression patterns were enhanced after MCD diet compared with control fed mice.

### Hepatocyte-specific *Cav1* knockout in NAFLD development

Although no phenotypic differences between HepCAV1ko and HepCAV1wt were observed under healthy conditions, transcriptomic alterations were determined. Thus, it was of interest to see whether the absence of hepatocytic *Cav1* was reflected on the gene expression level in diseased mice. Gene expression profiles of HepCAV1wt and HepCAV1ko upon feeding MCD diet were analyzed in context of gender.

In male MCD fed mice, 1000 genes were significantly regulated in HepCAV1ko compared to HepCAV1wt mice (in total 471 upregulated, including *Dmbt1, Fbl, Egr1*, and 529 downregulated genes, including *Evol3, Cyp2c40, Cyp2c38*; top 20 genes are listed in Fig. S5A, B). Upregulated genes were related to SNARE interactions in vesicular transport, amino sugar and nucleotide sugar metabolism. Downregulated genes were mainly involved in distinct metabolic processes (including arachidonic acid, tryptophan, and fatty acid metabolism, Fig. 6b). Biological process analysis confirmed the finding shown in pathway annotation with presenting lipid metabolic processes and oxidation-reduction processes (Fig. S5C). As conclusion, we report that male HepCAV1ko mice mainly responded with a downregulation of lipid metabolism-related genes upon MCD diet. However, on the phenotype level, this functional outcome did not manifest.

In females, 961 genes were deregulated (476 upregulated genes, including *Cyp3a16, A1bg, Trav9-2*, and 485 downregulated genes, including S*lco1a1, Gm6614, Ugt1a7c* in the knockouts, Fig. S6A, B). Deriving from KEGG pathway annotation, upregulated genes were related to metabolic pathways, e.g., carbon and amino acid metabolism, while downregulated genes were less clusterable in KEGG pathway, only showing cancer-relation, including microRNAs in cancer, viral carcinogenesis and prostate cancer (Fig. 6c). Biological process analysis and molecular function nevertheless supported the results from KEGG pathway annotation, showing metabolic processes and oxidoreductase activity as upregulated (Fig. S6C). Therefore, in females, HepCAV1ko induced metabolic processes while in males those were reduced. A few strongly altered metabolic genes from array analyses in males and females were tested by PCR. As in the array, *Elov3* and *Csad* were significantly changed, and for *Dpys* and *Got1* we found a clear trend (Fig. S7), supporting our array findings.

Deregulated metabolic genes in the HepCAV1ko were analyzed in four data subsets (male HepCAV1ko mice control diet vs wild-type, male HepCAV1ko mice MCD diet vs wild-type, female HepCAV1ko mice control diet vs wild-type, female HepCAV1ko mice MCD diet vs wild-type). As shown in Fig. 4b and Fig. S8, metabolic events were classified in diverse programs. Among them, small molecular transport, ion transport, fatty acid, glycan, and amino acid are most relevant metabolic processes. In context of the glucose metabolism relevance in liver regeneration^9^, we more closely analyzed whether genes related to glycometabolism were affected by diet (sex dependent). Functional involvement of altered genes in gluconeogenesis and glycolysis was rather low (~1–3% of metabolic altered genes). However, genes related to glycan metabolism were altered more frequent, with a share of 7–11% of metabolic altered genes (Table S9).

When overlapping all deregulated genes from males and females, only a small number of genes were found regulated in both sexes (Fig. S9, Table S10), indicating that CAV1 exerts gender-dependent effects in NAFLD disease.

To conclude, these results provide evidence that CAV1 prominently influences metabolic processes during disease development, but also determines gender effects.

## Discussion

In this study, we investigated the effects of hepatocyte-specific CAV1 in healthy and in NAFLD mice phenotype evaluation of tissue and gene expression profiling as well as plasma analysis.

The hepatocyte-specific knockout using the Cre/loxP system was successfully established, although mRNA expression of *Cav1* in liver tissue (*p* = 0.0649 in males, 0.0771 in females) did not reach significance, which is due to normal *Cav1* expression in non-parenchymal cells (and especially LSEC express higher levels compared to hepatocytes). In context of distinct cell types expressing higher CAV1 levels, this protein will exert cell specific functions and interestingly, in LSEC, CAV1 impacts on eNOS regulation, autophagy and defenestration—crucial events during liver diseases^18,19^. Therewith, applying the cell type specific knockout in context of LSEC and other liver cells will be relevant to broaden our understanding on CAV1 in liver disease contexts. Regarding parenchymal cells, the knockout in isolated hepatocytes was evident—as a significant reduction of *Cav1* was shown on RNA level in both sexes. On protein level, some CAV1 expression was still detectable in knockout hepatocyte lysates, likely due to the conditional knockout system that could be leaky. However, compelling evidence proves CAV1 reduction in the HepCAV1ko and thus, we moved onto study its functions in vivo.

CAV1 regulation in NAFLD showed conflicting results in the literature. CAV1 expression was repressed in L02 and AML12 cells cultured with free fatty acids to trigger steatosis in vitro. In vivo using C57BL/6 J mice fed a high fat diet (HFD) to induce NAFLD, CAV1 expression was reduced. Similar findings were obtained in obese mice, and even in NAFLD patients^10,20^. These findings are in line, showing a reduction of CAV1 expression upon feeding MCD in male mice, but not in females. However, in another study, C57BL/6 male mice fed a high fat and cholesterol enriched diet showed increased CAV1 expression in liver^21^. Regarding our study, CAV1 expression showed a tendency towards upregulation in MCD fed females. Hence, gender plays a crucial role in controlling CAV1 expression in NAFLD. Li et al. may provide a key explanation on the mechanism of CAV1 regulation in NAFLD: CAV1 expression was proved to be reduced by upregulated miR199a-5p in hepatocytes^20^. Indeed, this microRNA is regulated during liver diseases^22,23^. Thus, it is worth investigating whether miR199a-5p expression is gender-dependent.

The liver is pivotal in regulating systemic homeostasis and balancing metabolism, in concert with other tissue, e.g., adipose tissue and the gastrointestinal tract. In recent studies, CAV1 was proven as an important membrane protein for regulating metabolism and different signalling pathways, but most in vivo studies were done with global *Cav1* knockout animals, reporting some contradictory outcomes. For example, CAV1 was proven to protect from lipid accumulation in L02 and AML12 hepatocyte cells upon FFA treatment and in mice after 4 weeks HFD^10^. While in the Asterholm study, opposite results were shown, i.e., that *Cav1* knockout mice had reduced hepatic steatosis after 8 weeks HFD in response to 24 h fasting^13^. These conflicting results implicated that CAV1 abundance related steatosis may be influenced by diet/feeding conditions, for example whether animals were fasting prior analysis. They also assumed that the liver may play a secondary compensatory role as the metabolic dysfunctions should be mediated by adipose tissue^13^. These results were consistent with our findings using hepatocyte-specific *Cav1* knockout mice, pointing to the possibility that CAV1 in other tissues/cells affects susceptibility for steatosis. However, when studying liver regeneration upon partial hepatectomy, Fernández-Rojo and colleagues determined hepatocyte autonomous effects of CAV1^9^. *Cav1* knockout animals shifted towards carbohydrate-dependent adaptation. In AML12 cells, lack of CAV1 under metabolic stress led to alterations in the extracellular acidification rate, indicating alterations in lactate efflux and thus of glycolysis. The MCD NAFLD/NASH model is also triggering massive metabolic stress and hence, a similar adaptation might take place. With absent CAV1, glucose availability thus may influence hepatocyte integrity and proliferation capacity and therewith directly influence disease progression. Other animal models without lack of essential nutritional compounds, such as Western diet, might be more appropriate to study those mechanisms due to the mode of action. On gene expression level, data did not indicate major alterations of glycolysis or gluconeogenesis in dependency of CAV1. Comparing our analyses with GloCAV1ko mice data that were showing phenotypes like decreased body mass, increased liver/body ratio, increased triglyceride and cholesterol levels^13,24^, this was not observed in HepCAV1ko mice under control conditions. Also, comparing KEGG pathways and metabolic events of GloCAV1ko and HepCAV1ko mice, strong differences and little overlap were detected, thus consistent with the phenotypes.

Sexual dimorphism was discussed as second important factor in weight average proportion analysis. In this study, strong metabolic differences between the sexes were determined, regardless of genotype and diet. In previous studies, gender-specific metabolic differences caused by CAV1 were reported. In the CAV1 intestinal epithelial cell knockout mouse model, plasma low density lipoprotein (LDL) cholesterol increase by HFD was prevented in males, but not in females^25^. Also, LDL cholesterol was only detected in male knockout mice^11^. Here, we observed a GLDH gender difference in the HepCAV1ko mice upon MCD diet. More interestingly, male HepCAV1ko mice demonstrated suppressed, while females had induced metabolic gene expression patterns upon MCD feeding. However, strong basal differences (healthy conditions) between male and female FVB mice had been monitored (Table 2, Fig. 6a). Based on this, CAV1 seems to play sex-dependent metabolic functions in hepatocytes, although more studies are needed to determine in which contexts phenotypes become relevant. Regarding the mechanisms of CAV1 gender effects, some studies indicated that it may due to the interaction between CAV1 and sex hormone signaling to regulate metabolic processes, like androgen receptor^26^ or steroid signaling, such as E2^27^. Whether this regulation applies also in this setting has yet to be proven.

We identified significant alterations of gene expression profiles in HepCAV1ko mice in NAFLD, while not on the disease level. A possible reason for this is the short time feeding (4 weeks) which only presents the early phase of disease (with slight fibrosis). The HFD model could be applied to investigate later phases of NASH-disease for further studying effects of CAV1.

In summary, our findings provide evidence that hepatocyte-specific *Cav1* knockout does not affect MCD mediated NAFLD development, but alters expression level of a plethora of genes. *Cav1* deletion led to strong differences in gene expression profiles in male and female livers, in both healthy and NAFLD mice. Future studies will shed light on whether gender differences with respect to CAV1 function are of pathophysiological relevance in the development of metabolic liver diseases.

## Materials and methods

### Mice

Floxed CAV1^−/−^ mice were obtained from Prof. Philip Scherer (University of Texas) and details about the mice are depicted in the study of Asterholm et al.^13^. In this project, floxed CAV1^−/−^ mice were crossed with Albumin promoter Cre-recombinase (Alb-Cre) heterozygous mice (BL/6-TgN(Alb-Cre−/ + )) to obtain HepCAV1ko mice and wild-type littermates (HepCAV1wt). The mice genotype was confirmed by PCR of ear tissue using the following primers: floxed Cav1 (forward: 5′-3′: GTG CAT CAG CCG CGT CTA CTC and reverse: 5′-3′: GGC CGT AAC CTG AAT CTC TTC CCT TTG) and Alb-Cre PCR (forward: 5′-3′: AAG TTG AAT AAC CGG AAA TGG and reverse: 5′-3′: AGC TAC ACC AGA GAC GGA AAT). Mice were housed in a pathogen-free standard environment. Group allocation for the experiments was randomized and not blinded. Sample analyses were not blinded. All procedures with mice were performed according to national and international guidelines. Prior ethics approval was obtained from the local ethics committee of the state Baden-Württemberg.

### Experimental diet

Mice were kept with a normal diet ad libitum until 8 weeks of age. Prior start of the experiments, both HepCAV1ko and HepCAV1wt mice were randomly divided into two groups for either feeding the MCD diet (*N* = 12) or control diet with methionine and choline (*N* = 12) for 4 weeks. MCD and control diets were purchased from Ssniff (E15652 and E15654, Germany). Each diet group included 6 male and 6 female mice. Mice were weighted twice per week during the 4 weeks of experimentation.

### Serum analyses

At the end of the experiment, blood from retrobulbar plexus was preserved for further serum analyses. Blood biochemical indices, among alanine aminotransferase, aspartate aminotransferase (AST), alkaline phosphatase, glutamatdehydrogenase (GLDH), triglyceride, cholesterol, urea, and glucose were measured using a Hitachi automatic analyzer.

### Histological analysis

For histopathologic evaluation, livers were fixed with 4% paraformaldehyde, embedded in paraffin, and cut into 4 μm thick slides. Hematoxylin and eosin (H&E, Darmstadt, Germany) and Sirius red stainings (PSR, Missouri, USA) were done to assess liver inflammation and fibrosis. For detection of lipid droplets in livers, cryostat sections of 8 μm were stained with Oil Red O according to standard procedures. Sections were imaged at ×20 magnification. Quantification of Oil Red staining was done with ImageJ software (Version1.51j8). Immunohistochemistry for Ki67 (12202 S, Cell Signaling) was performed to determine cell proliferation activity in tissue of HepCAV1wt and HepCAV1ko mice fed normal or MCD diet. Quantification was done by counting positive nuclei upon ×20 magnification in 20 random fields per mouse. For each mouse, values were averaged and shown in the graph (Fig. S1).

### Hepatocyte isolation and culture

Primary hepatocytes were isolated with a two-step collagenase (C2-22, Merk Biochrom) perfusion method as described previously^28^. Freshly isolated hepatocytes were then seeded on 6-well plates (3 × 10^5^ per well) and cultured in Williams E medium supplemented with 1% l-glutamine, 1% penicillin/streptomycin, 0.1% dexamethasone, and 10% fetal bovine serum^28^. Upon attachment (approximately after 4 h), cells were cultured in starvation medium without dexamethasone and FBS. RNA and protein lysates of primary hepatocytes were harvested after 48 and 72 h.

### RNA isolation and quantitative PCR

Total RNA was isolated from whole liver tissue or primary hepatocytes using InviTrap Spin Universal RNA Mini Kit (Stratec Biomedical AG, Germany), according to the manufacturer’s instructions. RNA concentration was determined using the Tecan Infinite M200 microplate reader. Subsequently, RNA (1 µg) was reverse transcribed to cDNA using random primers and Revert Aid H Minus Reverse Transcriptase (Thermo Scientific). Quantitative polymerase chain reaction (qPCR) analysis was performed in a 10 µl total reaction volume using EvaGreen qPCR Mix Plus (Solis BioDyne, Tartu, Estonia) on a StepOne (Applied Biosystems). The qPCR reaction mix was composed of 2 µl EvaGreen, 12.5 ng cDNA, 5 pmol of gene primer mix, and then filled to 10 µl with nuclease-free water. The experiments were performed in triplicates (or as stated) and peptidylprolyl isomerase A (*Ppia*) was used for normalization. The gene primer sequences are as follows (5′-3′): *Cav1*: forward: GAA GGG ACA CAC AGT TTC GAC; reverse: GGA TGC CGA AGA TCG TAG ACA. *Ppia*: forward: GAG CTG TTT GCA GAC AAA GTT; reverse: CCC TGG CAC ATG AAT CCT GG. *Elovl3*: forward: TTC TCA CGC GGG TTA AAA ATG G; reverse: GAG CAA CAG ATA GAC GAC CAC. *Got1*: forward: GCG CCT CCA TCA GTC TTT G; reverse: ATT CAT CTG TGC GGT ACG CTC. *Dpys*: forward: GGT GGA GCG ACA AGG TAA AAG; reverse: CTG CTC GTC TTG CAC CAT GT. *Csad*: forward: GAA GAA GGG GAC CAT GAT GA; reverse: CAC CAC CAT TCG GAA GAA GT.

### Western blot analysis

CAV1 protein abundance was tested with tissue lysates from whole liver and isolated primary hepatocytes as previously described^29^. Briefly, RIPA buffer with freshly added phosphatase and protease inhibitors was used to extract protein from tissue and cells. Protein concentration was assessed with the Bio-Rad protein assay kit according to the manufacturer’s instructions and quantified with the Tecan Infinite M200 via absorbance measurement at 690 nm. Equal amounts of protein (30 µg) were separated by SDS-PAGE (10–12% gels) and blotted onto a PVDF membrane (Millipore, Billerica, MA, USA). The membrane was blocked with 5% nonfat dry milk in TBST at room temperature for 1 h, and incubated overnight at 4 °C with primary antibodies. Primary antibodies against CAV1 (T3267, Cell Signaling) and GAPDH (sc25778, Santa Cruz Biotechnology) were used. HRP-linked anti-mouse (sc-2005, Santa Cruz) and anti-rabbit antibodies (sc-2357, Santa Cruz) were used as secondary antibodies. Signals were visualized by incubating the blots in Supersignal Ultra solution (Pierce, Hamburg, Germany) and recorded with imaging system Fusion SL4 (PEQLAB, Germany). The target protein bands were quantified by using Fluorchem Q system (ProteinSimple, California, USA).

### Microarray analysis

Prior microarray analysis, RNA isolated from liver tissue was tested by capillary electrophoresis on an Agilent 2100 bioanalyzer (Agilent) for confirming quality. Gene expression profiling was subsequently performed using mouse Affymetrix MoGene 2.0 ST Array Chips (Life Technologies, Germany). Biotinylated antisense cRNA was then prepared according to the Affymetrix standard labelling protocol with the GeneChip WT Plus Reagent Kit and the GeneChip Hybridization, Wash and Stain Kit (both from Affymetrix, Santa Clara, USA). Afterwards, the hybridization on the chip was performed on a GeneChip Hybridization oven 640, then dyed in the GeneChip Fluidics Station 450 and thereafter scanned with a GeneChip Scanner 3000. All of the equipment used was from the Affymetrix Company (Affymetrix, High Wycombe, UK).

### Data analyses

In addition to our own microarray data, the dataset GSE35431 was downloaded from the Gene Expression Omnibus website (https://www.ncbi.nlm.nih.gov/geo/). This dataset included gene profiles of livers from global *Cav1* knockout (GloCAV1ko) and wild-type littermates (GloCAV1wt). A *p* value < 0.05 was defined as statistical significant. The significantly regulated genes were followed by Kyoto Encyclopedia of Genes and Genomes (KEGG) pathway functional annotation and gene ontology analysis with DAVID Bioinformatics Resources 6.8 (https://david.ncifcrf.gov/). Venny tool was used to identify overlapping genes in two or more datasets (http://bioinfogp.cnb.csic.es/tools/venny/). Metabolic genes were defined as published in another gene list^30^.

### Statistics

Statistical analyses were performed with GraphPad Prism version 6.0 software. Statistical testing was done with two sided *t*-test or analysis of variation (ANOVA) where applicable. Normally distributed data was analyzed with *t*-test and non-normally distributed data was analyzed with Mann-Whitney test. We used ANOVA with multiple comparisons and Tukey’s posthoc test. Quantification of staining was done using ImageJ. Data is presented as mean ± SD. Each experiment was performed at least three times (or as indicated). Statistical significance is indicated as follows: *p < 0.05; **p < 0.01; ***p < 0.001. ****p < 0.0001

## Supplementary information

Supplementary figure and table legends

Figure S1

Figure S2

Figure S3

Figure S4

Figure S5

Figure S6

Figure S7

Figure S8

Figure S9

Table S1

Table S2

Table S3

Table S4

Table S5

Table S6

Table S7

Table S8

Table S9

Table S10
