# Supplementary material for: Hepatocyte caveolin-1 modulates metabolic gene profiles and functions in non-alcoholic fatty liver disease
Source: Cell Death Dis. 2020 Feb 6;11(2):104. doi: 10.1038/s41419-020-2295-5 (PMC7005160; doi:10.1038/s41419-020-2295-5)
Supplement: Supplementary file 1 — Supplementary figure and table legends [file 41419_2020_2295_MOESM1_ESM.docx]

**Supplementary information**

**Supplementary Figure Legends (Figure S1-S9)**

**Figure S1.** Ki67 staining in livers of HepCAV1ko and HepCAV1wt mice upon feeding MCD diet for 4 weeks. No significant differences in Ki67 staining between HepCAV1ko and HepCAV1wt mice with control diet or MCD diet (N=3 per group).

**Figure S2.** Microarray analysis of livers from male HepCAV1ko vs HepCAV1wt mice. (A) Number of differentially regulated genes in male HepCAV1ko livers (N=1211). (B) List of top 20 up- and downregulated genes. (C) Ontology analyses of deregulated genes. ↑: upregulated, ↓: downregulated; red: upregulated, green: downregulated.

**Figure S3.** Gender differences of deregulated genes in males compared to females. KEGG pathway annotation of highly or lowly regulated genes comparing males to females in HepCAV1ko mice with control diet (A), in HepCAV1wt mice with MCD diet (B), in HepCAV1ko mice with MCD diet (C) ↑: upregulated, ↓: downregulated; M: males, F: females.

**Figure S4.** (A) Overlap of 382 deregulated metabolic genes (male vs female HepCAV1wt mice) and 433 (male vs female HepCAV1ko mice) fed control diet (WT Con (M vs F) and KO Con (M vs F)). (B) Overlap of 204 deregulated metabolic genes (male vs female HepCAV1wt mice ) and 239 (male vs female HepCAV1ko mice ) fed MCD diet (WT MCD (M vs F) and KO MCD (M vs F)).

**Figure S5.** Changes in gene expression after MCD diet in HepCAV1ko compared to HepCAV1wt male mice. (A) Number of differentially regulated genes (N=1000). (B) List of top and bottom 20 significantly regulated genes. (C) Gene ontology analyses of up- or down-regulated genes confirmed the results from KEGG pathway determination.

**Figure S6**. Gene expression alterations of *Cav1* knockout hepatocytes relative to HepCAV1wt female mice upon MCD diet. (A) Number of differentially regulated genes (N=961). (B) Top and bottom 20 significantly regulated genes. (C) Gene ontology analyses of up- or down-regulated genes showed the same result with KEGG pathway annotation.↑: upregulated, ↓: downregulated; red: upregulated, green: downregulated.

**Figure S7.** qPCR analysis of deregulated metabolic genes in male and female HepCAV1ko mice compared with HepCAV1wt mice upon MCD diet. *Elov3* and *Csad* were significantly changed as found in the microarray data, and *Dpys* and *Got1* showed a clear trend of regulation.

**Figure S8.** Sector graphs for deregulated metabolic genes between HepCAV1ko and HepCAV1wt, sex and diet dependent. (A) Male control diet (KO vs WT). (B) Male MCD diet (KO vs WT). (C) Female control diet (KO vs WT). (D) Female MCD diet (KO vs WT). Small molecular transport, ion transport, fatty acid, glycan and amino acid are the most relevant altered metabolic events within the four datasets.

**Figure S9.** Overlap of 1000 genes deregulated by HepCAV1 knockout in male mice and 961 genes deregulated by HepCAV1 knockout in female mice fed the MCD diet (Male MCD (KO vs WT) and Female MCD (KO vs WT)).

**Supplemantary Table Legends (Table S1-S10)**

**Table S1.** Top 100 up- or downregulated genes by HepCAV1ko in male mice with control diet.

**Table S2.** KEGG pathway annotation of genes deregulated in HepCAV1ko vs HepCAV1wt.

**Table S3.** KEGG pathway annotation of genes deregulated in GloCAV1ko vs GloCAV1wt.

**Table S4.** Top 100 up- or downregulated genes by MCD diet in HepCAV1wt male mice.

**Table S5.** KEGG pathway annotation of genes deregulated by MCD diet in HepCAV1wt male mice.

**Table S6.** Top 100 up- or downregulated genes in males vs females in HepCAV1wt mice with control diet.

**Table S7.** KEGG pathway annotation of deregulated genes in males vs females in HepCAV1wt mice with control diet.

**Table S8.** Overlapping genes between males and females.

**Table S9.** Glycometabolic genes regulated by genotype, gender, diet.

**Table S10.** Overlapping genes between Male MCD (KO vs WT) and Female MCD (KO vs WT).
