# Supplementary figures and images for: Hepatocyte caveolin-1 modulates metabolic gene profiles and functions in non-alcoholic fatty liver disease
Source: Cell Death Dis. 2020 Feb 6;11(2):104. doi: 10.1038/s41419-020-2295-5 (PMC7005160; doi:10.1038/s41419-020-2295-5)

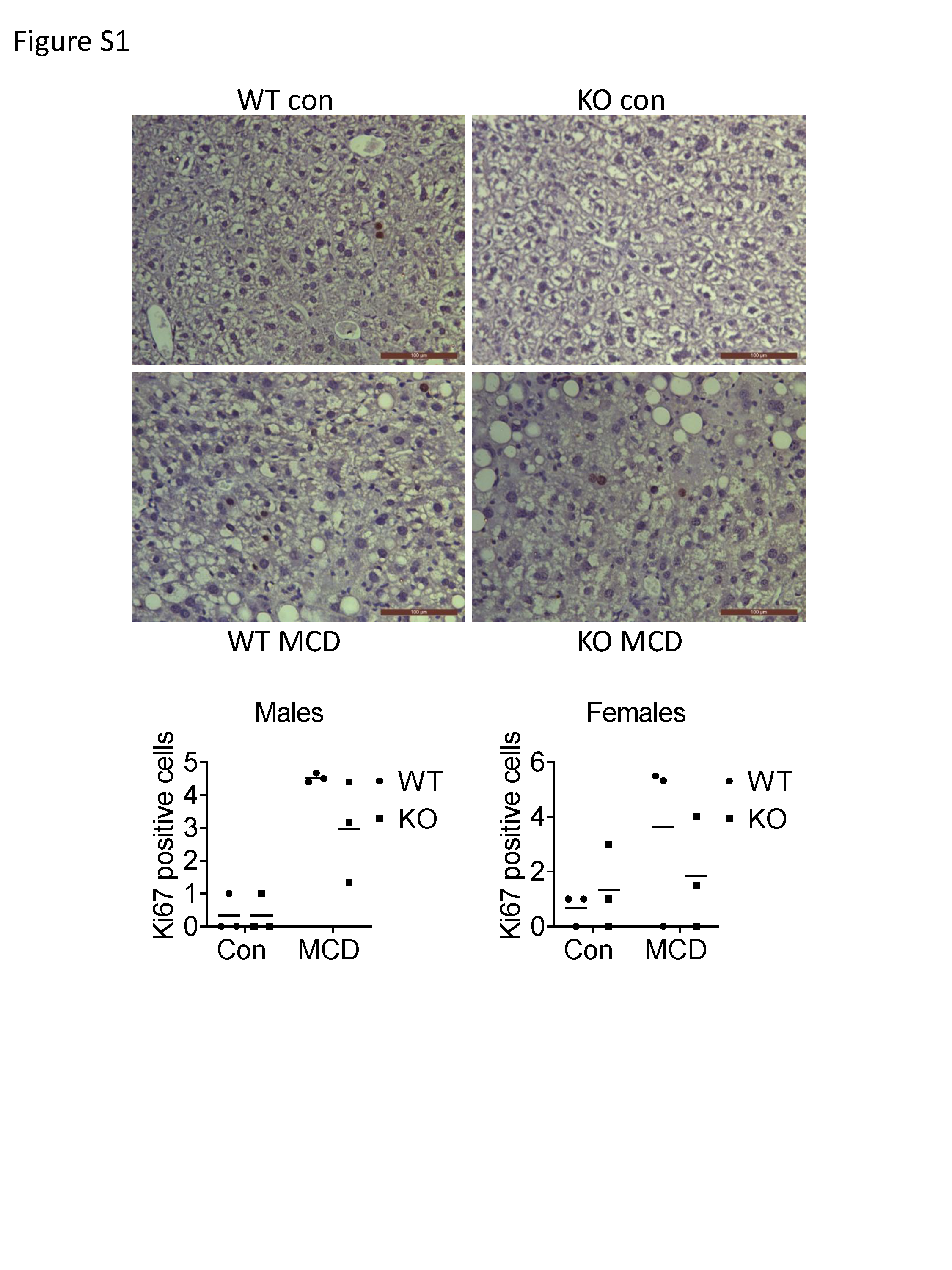

Supplement: Supplementary file 2 — Figure S1 [file 41419_2020_2295_MOESM2_ESM.png]

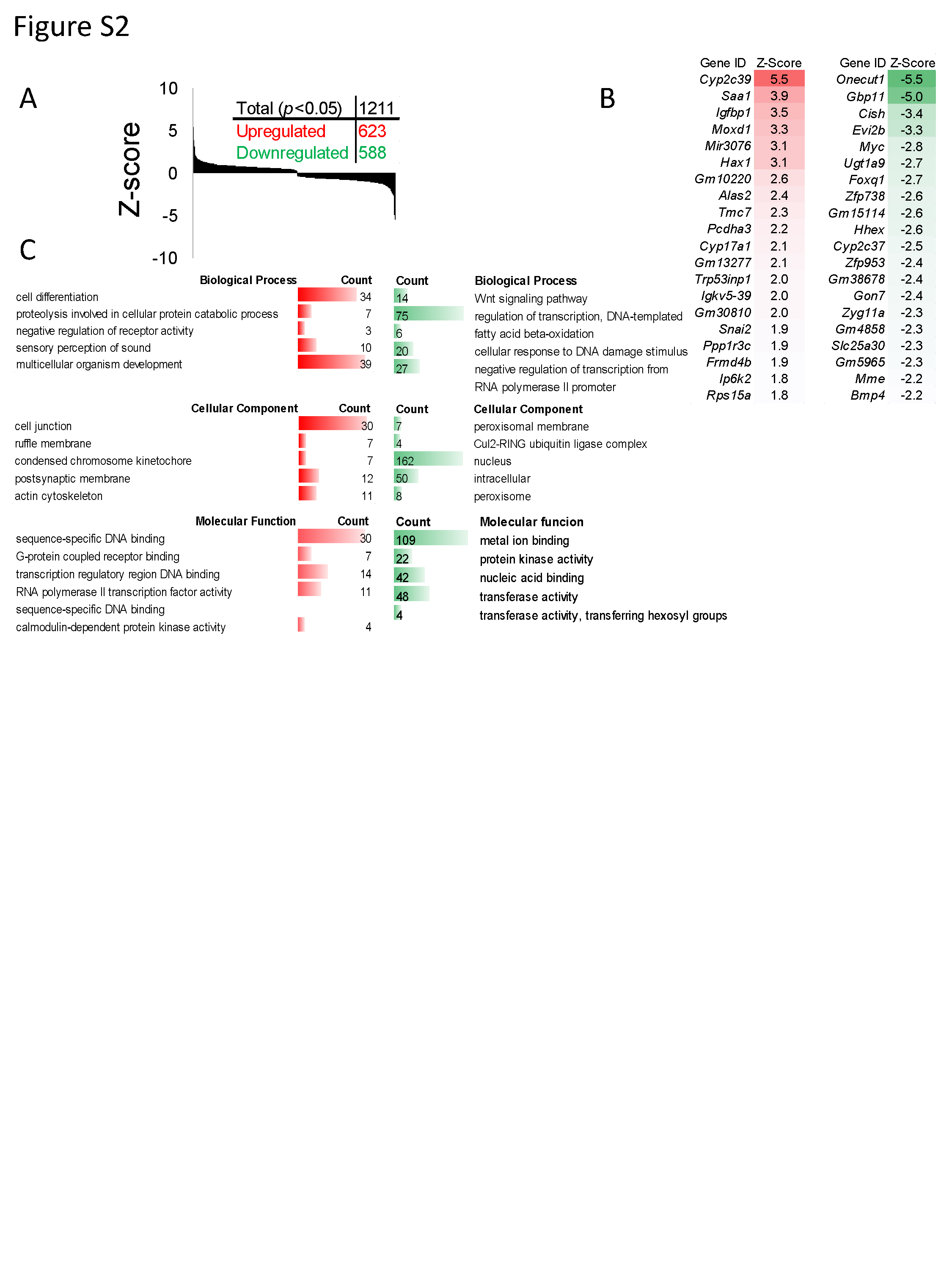

Supplement: Supplementary file 3 — Figure S2 [file 41419_2020_2295_MOESM3_ESM.png]

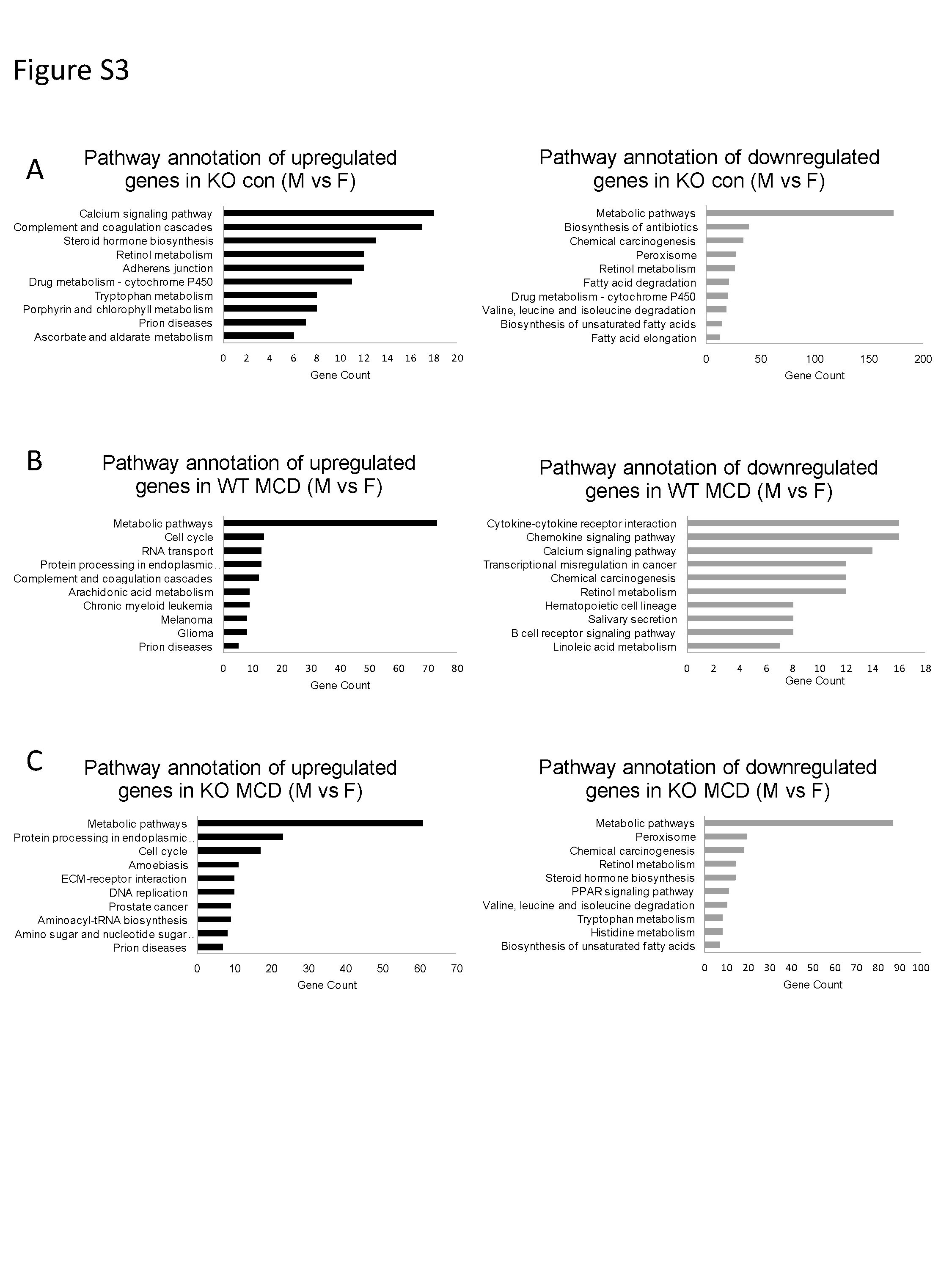

Supplement: Supplementary file 4 — Figure S3 [file 41419_2020_2295_MOESM4_ESM.png]

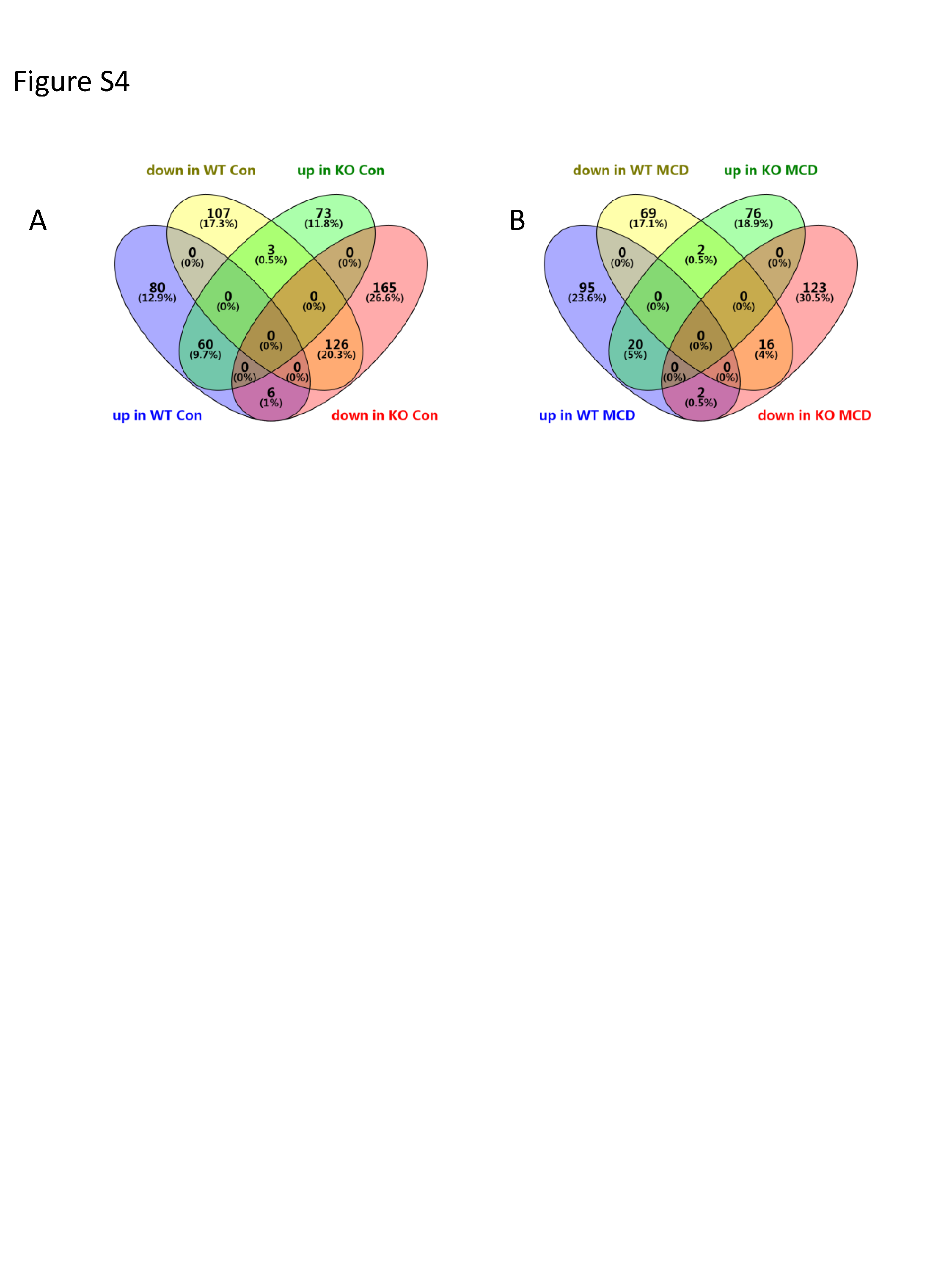

Supplement: Supplementary file 5 — Figure S4 [file 41419_2020_2295_MOESM5_ESM.png]

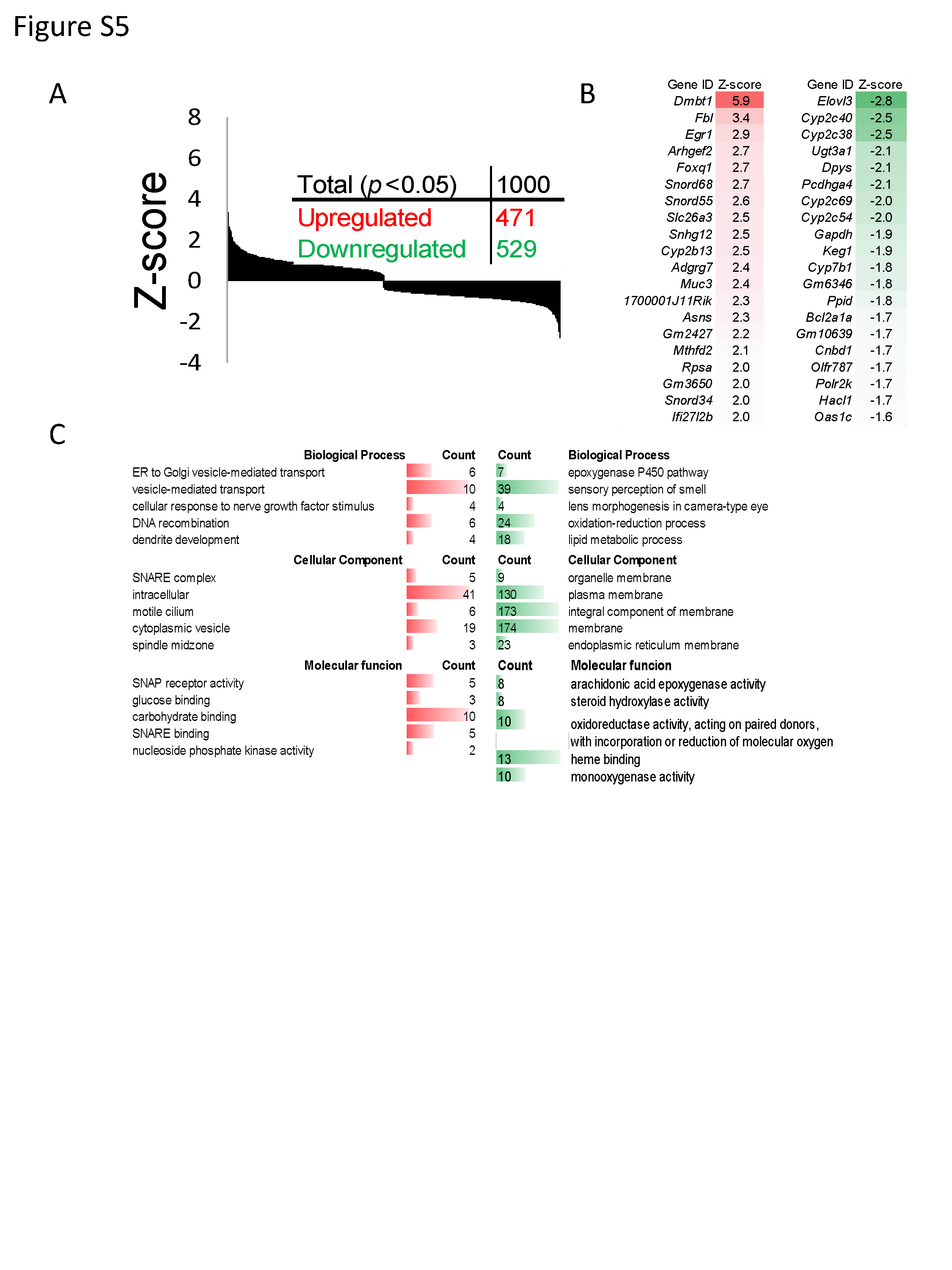

Supplement: Supplementary file 6 — Figure S5 [file 41419_2020_2295_MOESM6_ESM.png]

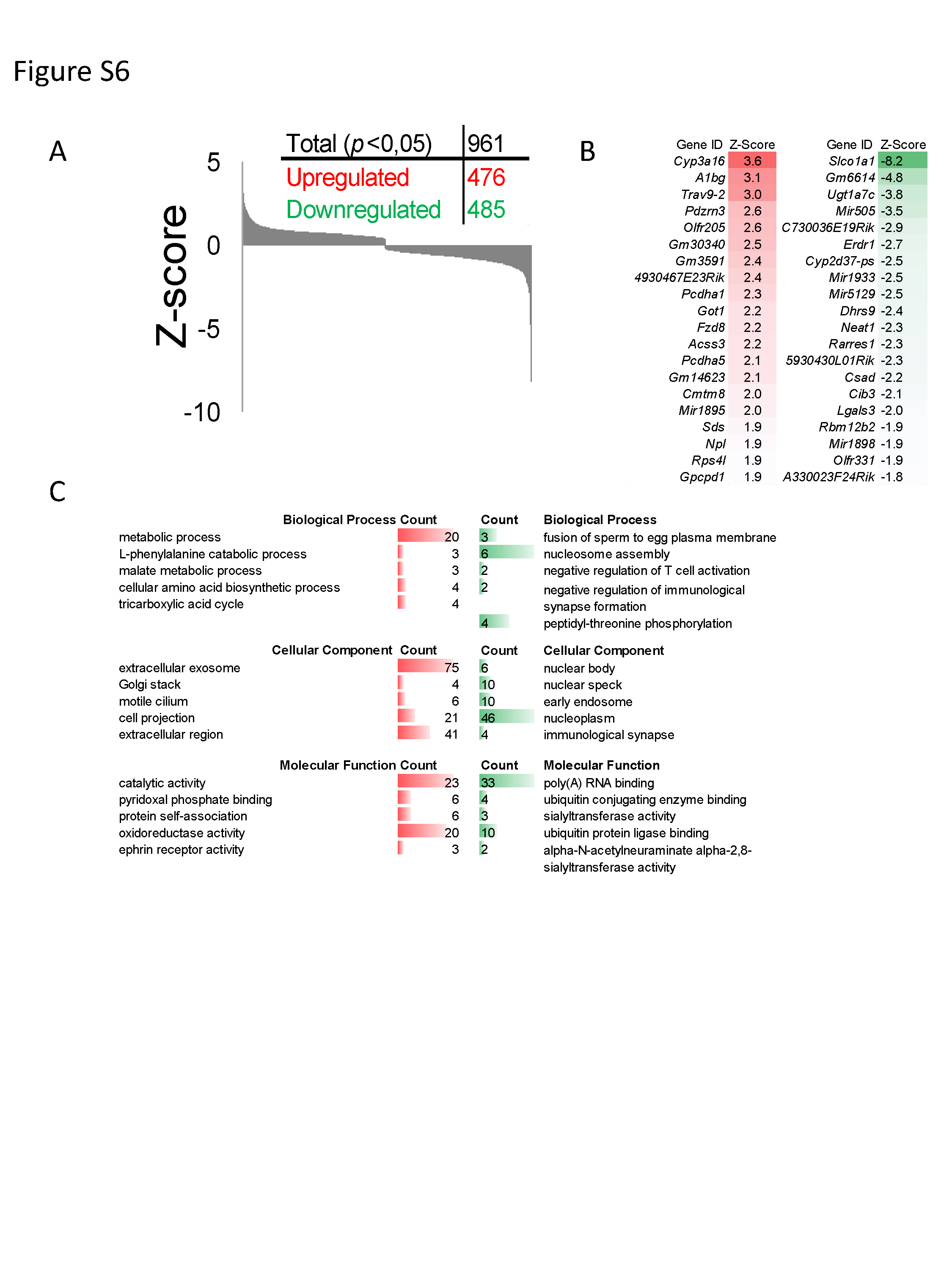

Supplement: Supplementary file 7 — Figure S6 [file 41419_2020_2295_MOESM7_ESM.png]

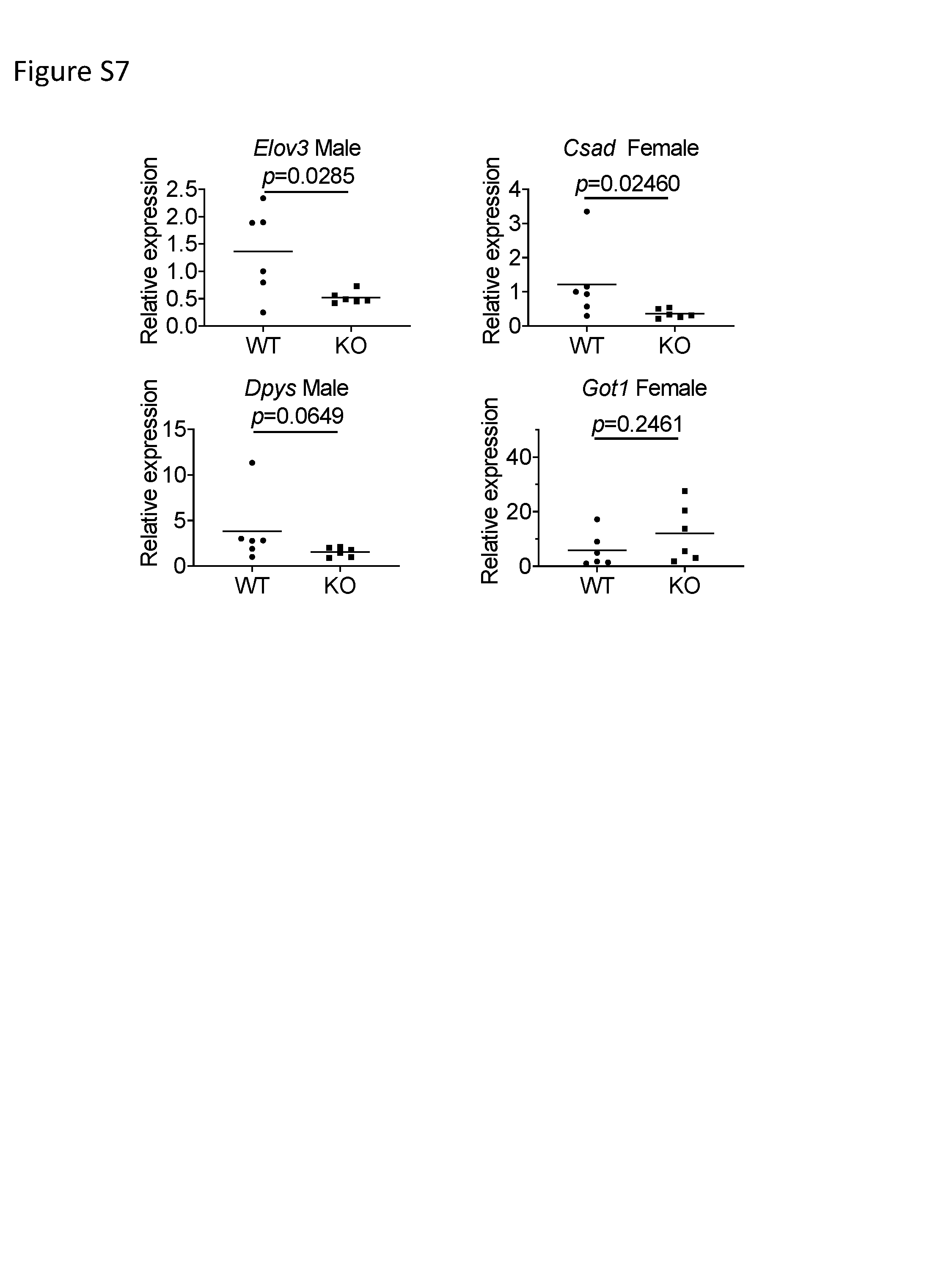

Supplement: Supplementary file 8 — Figure S7 [file 41419_2020_2295_MOESM8_ESM.png]

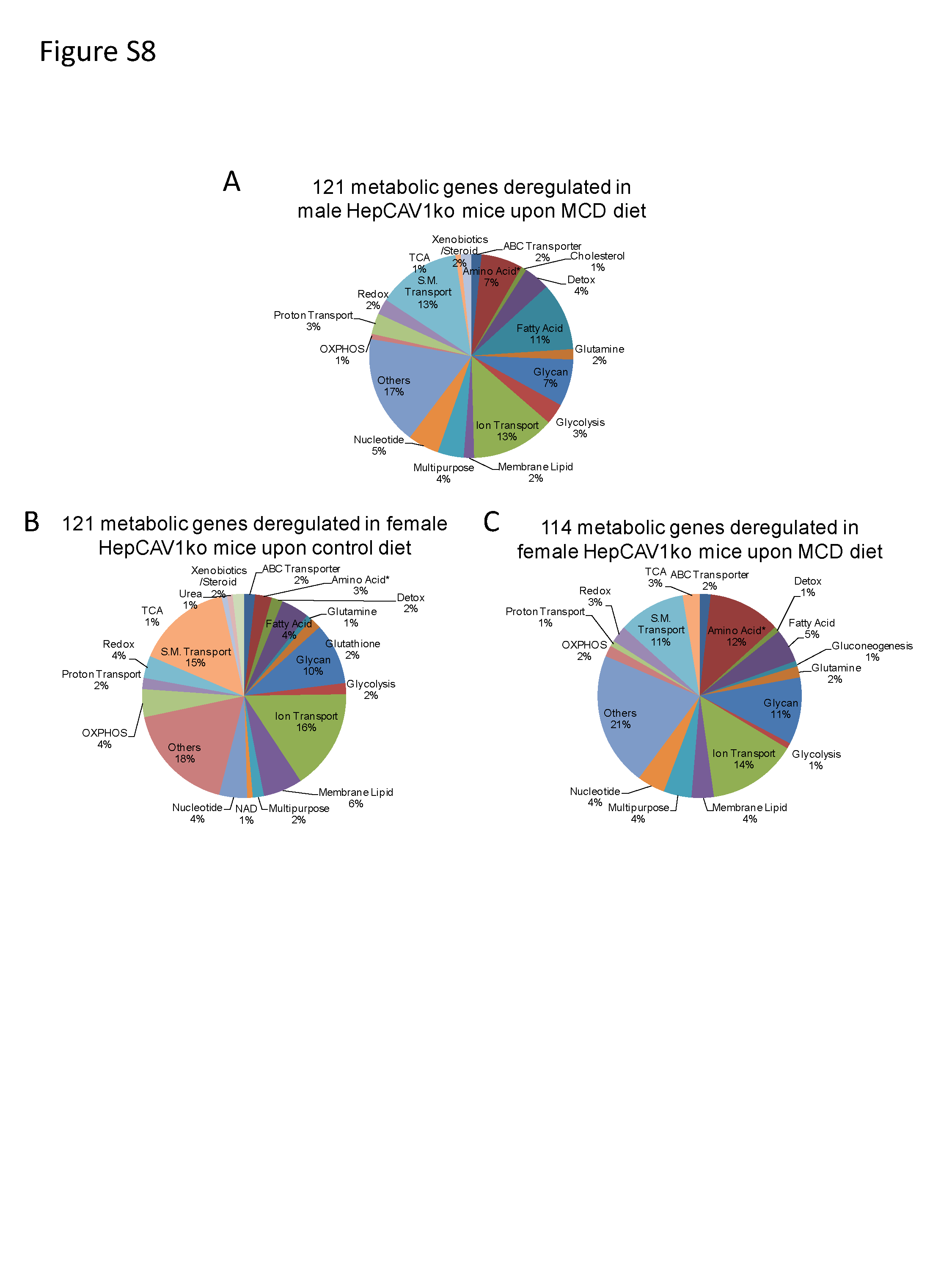

Supplement: Supplementary file 9 — Figure S8 [file 41419_2020_2295_MOESM9_ESM.png]

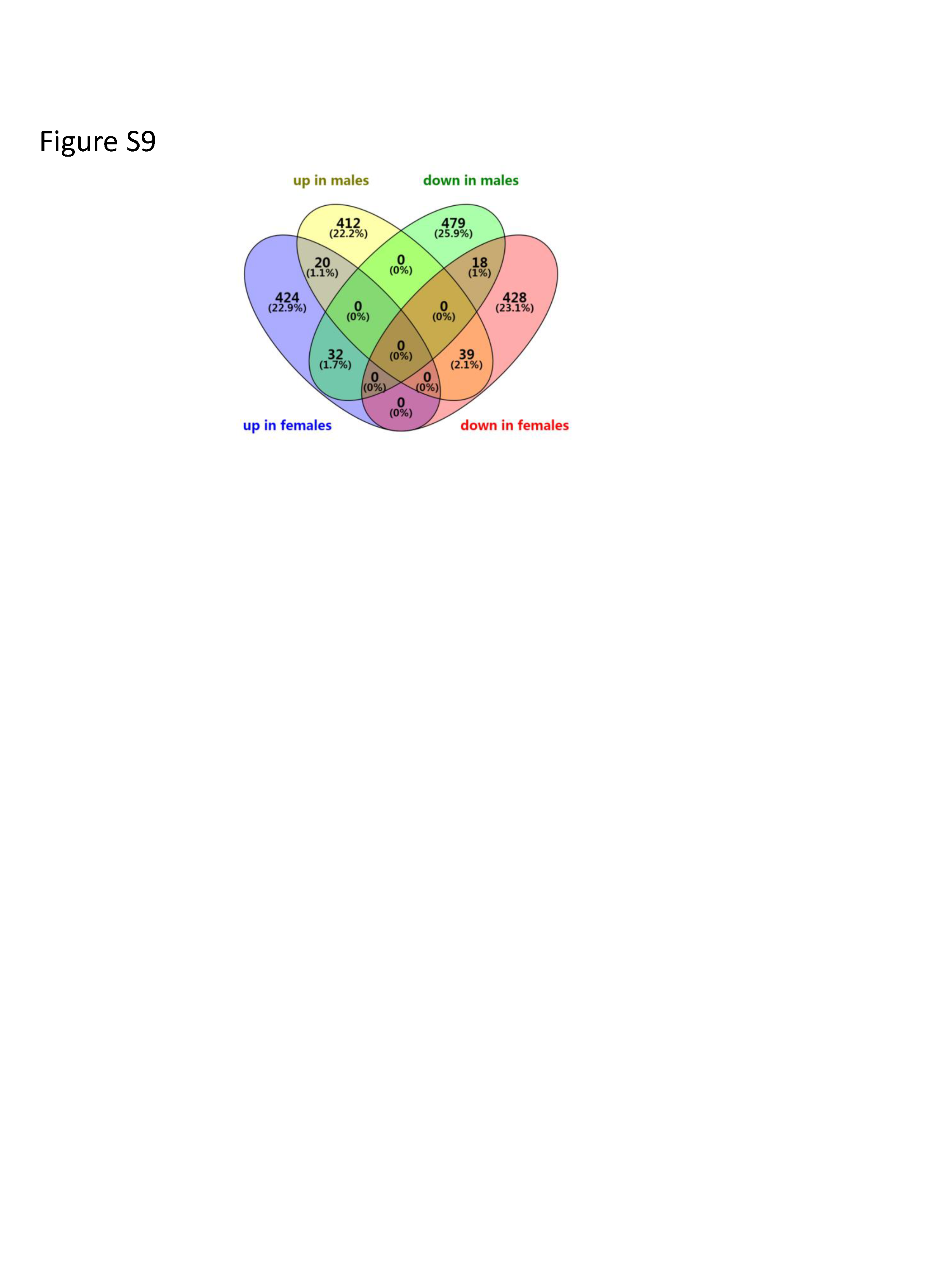

Supplement: Supplementary file 10 — Figure S9 [file 41419_2020_2295_MOESM10_ESM.png]
